# Intracranial Pressure Monitoring Signals After Traumatic Brain Injury: A Narrative Overview and Conceptual Data Science Framework

**DOI:** 10.3389/fneur.2020.00959

**Published:** 2020-08-28

**Authors:** Honghao Dai, Xiaodong Jia, Laura Pahren, Jay Lee, Brandon Foreman

**Affiliations:** ^1^Department of Mechanical and Materials Engineering, College of Engineering and Applied Sciences, Cincinnati, OH, United States; ^2^NSF I/UCRC Center for Intelligent Maintenance Systems, Cincinnati, OH, United States; ^3^Department of Neurology and Rehabilitation Medicine, University of Cincinnati College of Medicine, University of Cincinnati Gardner Neuroscience Institute, Cincinnati, OH, United States

**Keywords:** data science, intracranial pressure, traumatic brain injury, machine learning, prognostics and health maintenance

## Abstract

Continuous intracranial pressure (ICP) monitoring is a cornerstone of neurocritical care after severe brain injuries such as traumatic brain injury and acts as a biomarker of secondary brain injury. With the rapid development of artificial intelligent (AI) approaches to data analysis, the acquisition, storage, real-time analysis, and interpretation of physiological signal data can bring insights to the field of neurocritical care bioinformatics. We review the existing literature on the quantification and analysis of the ICP waveform and present an integrated framework to incorporate signal processing tools, advanced statistical methods, and machine learning techniques in order to comprehensively understand the ICP signal and its clinical importance. Our goals were to identify the strengths and pitfalls of existing methods for data cleaning, information extraction, and application. In particular, we describe the use of ICP signal analytics to detect intracranial hypertension and to predict both short-term intracranial hypertension and long-term clinical outcome. We provide a well-organized roadmap for future researchers based on existing literature and a computational approach to clinically-relevant biomedical signal data.

## Introduction

Traumatic brain injury (TBI) is one of the most common forms of acquired brain injury (1). While the primary injury associated with brain trauma consists of focal hematomas, contusions, and diffuse injury, secondary injury occurs once the patient has survived to hospitalization. Secondary injuries include cellular damage, inflammation, changes in the regulation of blood flow and, as the brain tissue begins to swell, elevations in the pressure exerted on the contents of the intracranial space, or intracranial pressure (ICP). ICP measurement is a cornerstone of physiologic neuromonitoring after brain injury in neurocritical care, and the use of ICP measurement after severe TBI, in particular, is thought to act as a biomarker of secondary brain injury. Guidelines provide Class II recommendations for the use of ICP monitoring after severe TBI (2) and targeted treatment to reduce ICP is thought to have a positive impact on long-term functional outcome.

## Methods

We performed a narrative overview of existing literature. The objectives of this overview were to discuss the existing approaches to the study of ICP signals from the perspective of data science and to highlight existing work related to signal processing and computational analytics organized around a conceptual framework. All studies were selected based on the expertise of the authors in order to highlight concepts important to the objectives of this overview.

## Discussion

### Background: The Intracranial Pressure Signal

The intracranial space is generally composed of three elements: 80% brain tissue, 12% venous and arterial blood, and 8% cerebrospinal fluid (3). The Monro-Kellie hypothesis familiar today was crystallized by Harvey Cushing (4, 5) and can be formulated as follows:

$$\begin{array}{l} V_{intracranial\;vault}=V_{brain}+V_{blood}+V_{CSF} \end{array}$$

This indicates that the volume of the intracranial contents *V*_*intracranial vault*_ is the sum of volumes of brain *V*_*brain*_, cerebrospinal fluid (CSF) *V*_*CSF*_, and the intracranial blood *V*_*blood*_ in the cranial cavity and is constant or nearly constant. An increase in any of these three should cause a decrease of the other two. The circulatory CSF component can be expressed by Davson's equation (6):

$$\begin{array}{l} \text{ICP}\left(\text{CSF}\right)=R\times F+P \end{array}$$

where *R* is the resistance to CSF outflow, *F* is the CSF formation and *P* is the pressure in sagittal sinus. Under pathological conditions, such as after TBI, a variety of mechanisms may cause an increase in ICP including an increase in brain volume (e.g., cerebral edema), an increase in arterial blood volume related to disruption in autoregulation, or obstruction of normal CSF circulation and absorption. The perfusion pressure within brain tissue capillaries is estimated by the following:

$$\begin{array}{l} \text{CPP}=\text{MAP}-\text{ICP} \end{array}$$

where CPP is the cerebral perfusion pressure and MAP is the mean arterial (input) pressure, and ICP functions as the resistance. Thus, elevations in ICP may lead to a decrease in CPP. As CPP declines and the lower limits of the brain's autoregulatory capacity are reached, cerebral blood flow reduces, and ischemia may develop depending on the metabolic needs of the tissue.

**Figure 1:**
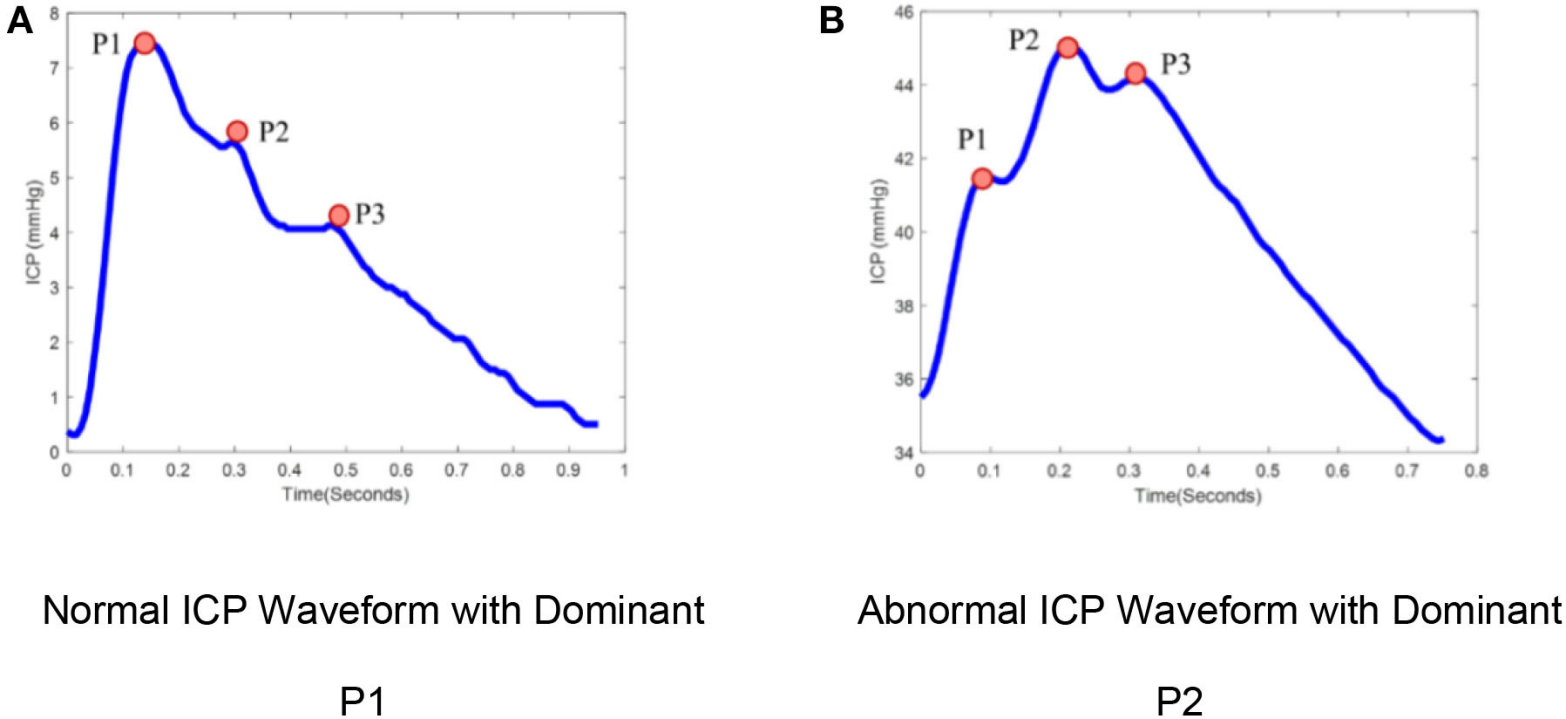
ICP pulse morphology: **(A)** Normal ICP Waveform with dominant P1, **(B)** Abnormal ICP waveform with dominant P2. ICP waveforms during normal intracranial physiologic conditions **(A)** differ from waveforms recorded when brain compliance is reduced **(B)**. The waveform **(A)** represents the dominant P1 with amplitude *P*1 > *P*2 > *P*3 while waveform **(B)** represent the dominant P2 with amplitude *P*2 > *P*1, *P*3.

ICP is typically measured either by catheters placed into the ventricular system, creating a closed fluid column, or using mini-strain gauge probes placed directly into brain tissue. ICP measured at adequate sampling frequencies (usually 64 Hz or greater) exhibits a characteristic waveform with three peaks, reflecting cerebral arterial pulsations with contributions from the venous compartment (7, 8). The three sub-peaks are referred to as percussion (P1), tidal (P2), and dicrotic waves (P3; Figure 1). ICP waveforms during *normal* intracranial physiologic conditions (Figure 1A) differ from waveforms recorded when brain compliance is reduced (Figure 1B) (9). Therefore, the pulse morphology contains useful clinical information, but quantification is challenging without the aid of advanced technologies (10).

**Figure 2:**
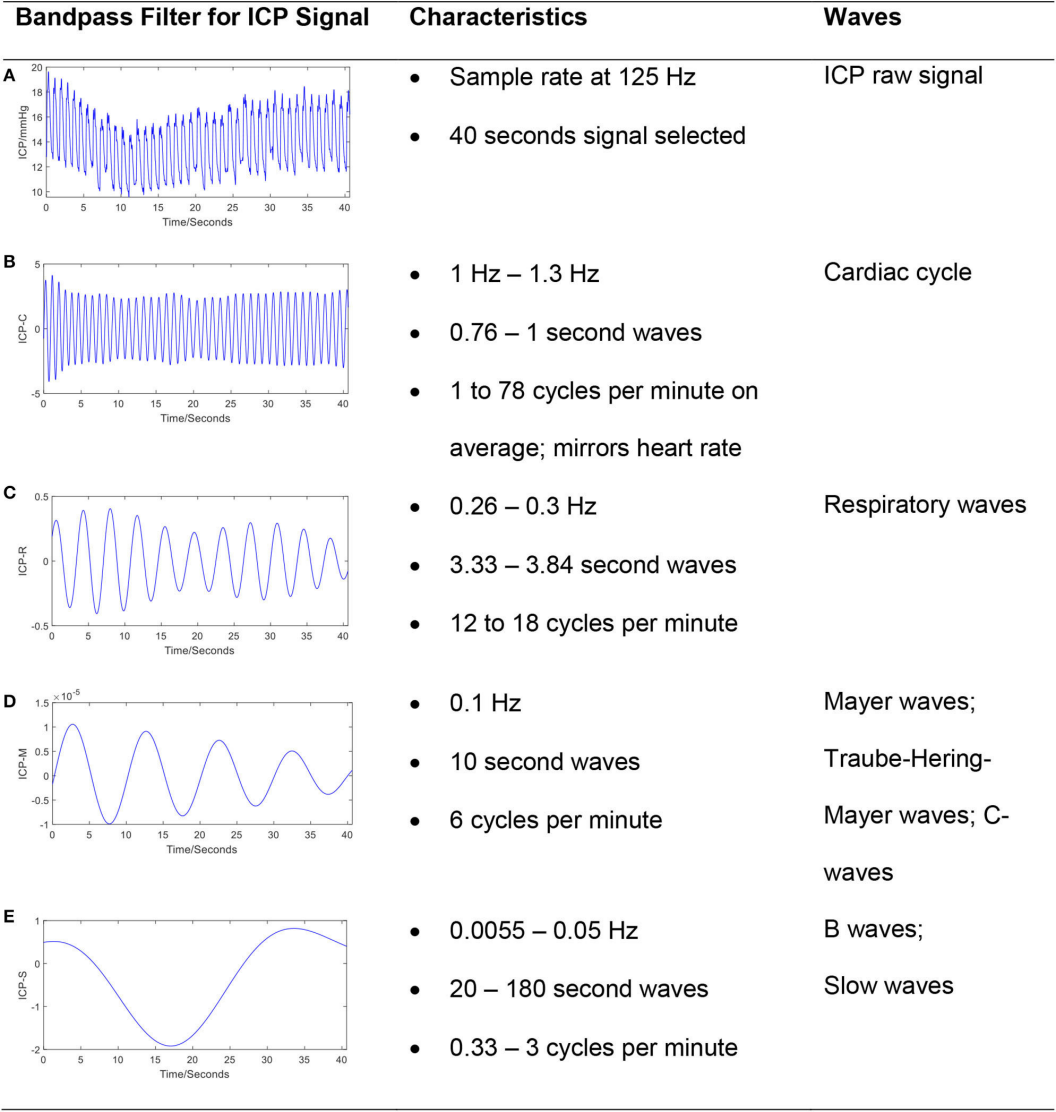
The frequency components in a typical ICP signal. The ICP signal can be decomposed into frequency domain components based on contributing sources. The bandpass filter technique has been applied to raw ICP signal **(A)** to extract cardiac cycle **(B)**, respiratory waves **(C)**, Mayer's waves **(D)**, and slow waves **(E)**. The passband frequency ranges (Hz) for bandpass filter is set to [1, 1.3], [0.26, 0.3], [0.1, 0.101], and [0.0055, 0.5], respectively.

In addition to the pulse component of the ICP waveform, the ICP signal also oscillates over slower timescales. The ICP signal can be decomposed into frequency domain components based on contributing sources (8, 11, 12) (Figure 2). The pulse waveform described above is related to the cardiac cycle and has a frequency band of 1–1.3 Hz. Mayer's waves (analogously termed C-waves) have a frequency of ~0.1 Hz, and are potentially associated with sympathetic nervous activity involved in the regulation of blood pressure (13). Respiratory waves are related to the respiratory cycle and oscillate between 0.26 and 0.3 Hz. Finally, B-waves have been defined by a frequency range between 0.33 and 3 cycles per minute, may be associated with fluctuations in cerebral blood volume (14–16), and can be stratified by amplitude (17).

Despite the richness and complexity of the ICP signal, current clinical measurements are reported almost exclusively as a simple average of the peak ICP over a short time window. Management is driven by medications or physiologic manipulation aimed at reducing the mean ICP below a set threshold deemed to be critically elevated. By attempting to maintain an ICP below a critical threshold, clinicians hope to decrease the likelihood of unfavorable outcome. However, the value of this threshold is not definitively established. Historically, studies have suggested mean ICP thresholds between 15 and 25 mmHg should be used (18–20). The most recent recommendation from the Brain Trauma Foundation is a threshold of 22 mmHg, largely stemming from a study comparing the average ICP over a 5 days period in a cohort of patients with TBI and demonstrating an association with increased mortality when the average ICP was above this threshold (2).

One size may not fit all. It seems likely that ICP thresholds for a given individual vary with personal characteristics (e.g., age, sex, medical history, and treatment intervention) (21). A retrospective cohort study of 355 patients concluded that critical ICP thresholds also vary with time and may exists below 20 mmHg after the 1st day of monitoring (22). Finally, TBI with mass lesions or elevations in ICP may require decompressive hemicraniectomy (DC) (23), a surgery which effectively reduces intracranial pressure while limiting the assumptions of the Monro-Kellie doctrine. In a meta-analysis of 590 participants in trials designed to evaluate the use of decompression to treat ICP-related morbidity and mortality, effective reduction in ICP *via* DC was *not* associated with an overall improvement in “favorable outcome,” although the relative risk of death and/or the minimally conscious state at 1 year following injury was reduced significantly (24). The targeted treatment of ICP based on threshold values may be rooted in reasonable inference, but may reflect an overly simplistic approach to the intracranial compartment's complex physiology. ICP is crucially linked to other factors such as cardiac output and arterial blood pressure, the effects of carbon dioxide tension on cerebral vasculature, and the delivery of oxygen and glucose to injured tissue. However, a first step to understanding the relationships between ICP and other physiologic parameters requires a comprehensive characterization of the ICP signal itself.

### Conceptual Data Science Framework

**Figure 3:**
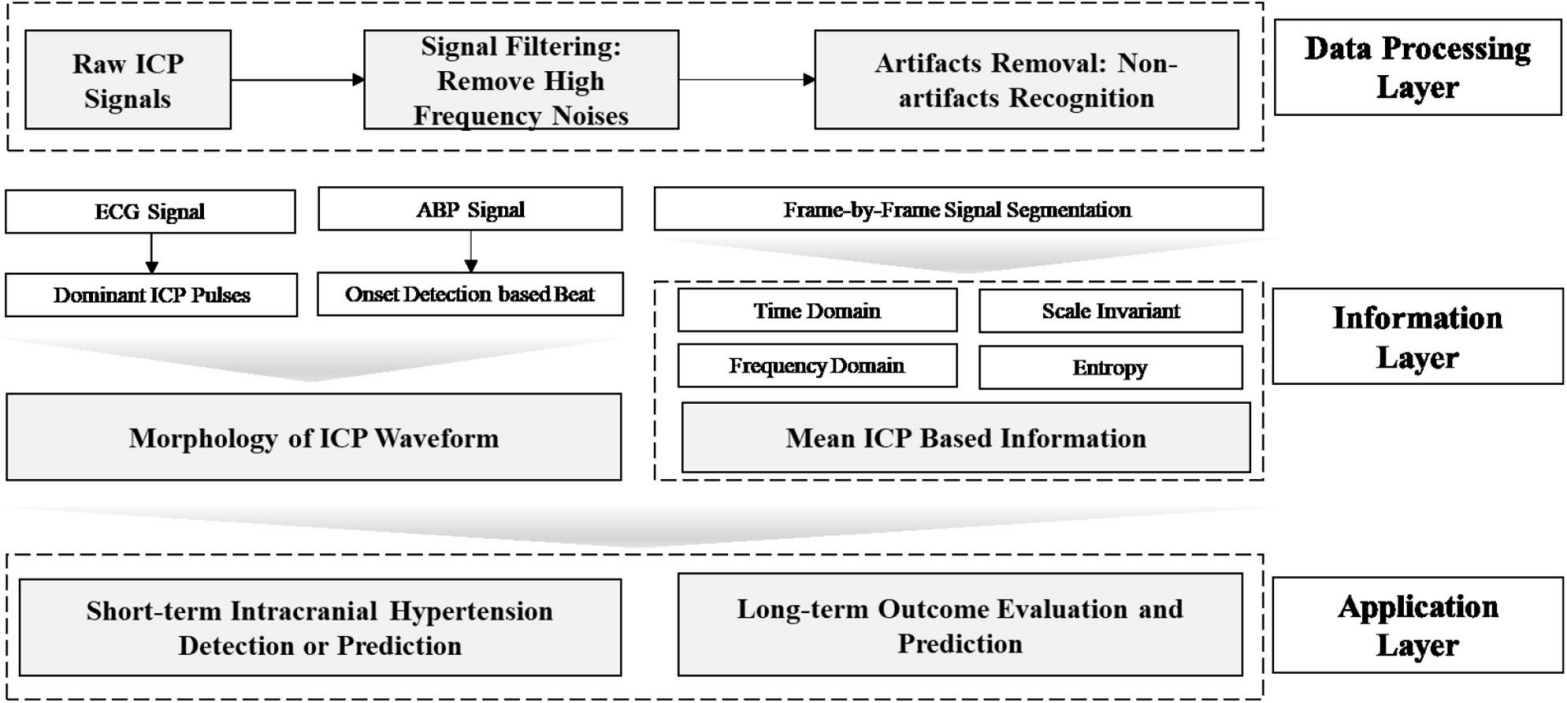
Framework of data mining approaches for ICP signals in patients with TBI. In this framework, the raw ICP signal is fed into a data processing layer to remove high frequency noise and artifacts. Then, features are extracted in the information layer, across two major categories: morphology and segment signal characteristics, including time and frequency domain features. In order to extract the morphology of the ICP waveform, ECG or ABP serve as reference signal to help identify ICP pulse waves and peak detection can validate the accuracy of extracted features. Final, an application layer provides a link between the ICP waveform and clinically relevant events.

Data mining approaches are ideal to quantify the multiple signal characteristics of ICP over time in order to achieve a more comprehensive understanding of the role of ICP in brain monitoring (Table 1). A proposed framework for a data mining approach to the ICP signal is presented in Figure 3. This framework is formulated as an engineering problem in order to solve biomedical questions using data science and will guide the following review of the literature on this subject.

**Table 1:** Main strength and limitation of techniques summary.

**Methods**	**Techniques**	**Strength**	**Limitation**
Processing: signal filtering	Low-pass filters	Remove small amount of high frequency noise when cutoff frequency can be explicitly set	Need to customize filter design for better effects
Processing: artifact removal	Signal decomposition	Recover the underlying process of ICP signal; efficient, and easy to implement in real time	Threshold may be difficult to set at patient-level
	Dominant ICP pulse	Robust to various artifacts due to averaging and retains the morphology of major ICP clusters	Remove useful signal potentially
Information extraction	Mean ICP features	Robust to artifacts and easy to implement in real time	Unable to detect ICP pulse waveform abnormalities
	Morphology features	Tracks and characterizes ICP morphology	Sensitive to artifacts; requires reference library. Extraction may require additional signals (e.g., ECG)
Modeling application	Statistical modeling	Explicitly infer the significance and relationship of variables between control and treatment groups	Require pre-define rules
	Machine learning	Make the most accuracy prediction as possible and easy to implement in real time	Can be black box and hard to explain

A variety of non-invasive techniques have been described to facilitate ICP monitoring without the risks associated with ventricular or parenchymal catheters, such as symptomatic hemorrhage (25). At their simplest, non-invasive measures merely dichotomize critical thresholds of ICP. Optic nerve ultrasonography measures the diameter of the nerve sheath, which is in continuity with the subarachnoid space and therefore increases when ICP is critically elevated (26). Other approaches rely on intermittent measurements, such as CT or MRI imaging (27). Intermittent Doppler-based techniques have been used to insonate vessels exposed to pressures within the skull. Recently, the use of transcranial Doppler fixation devices has allowed for the relatively continuous assessment of mean middle cerebral artery blood flow velocity (CBFV) in patients who are sedated or comatose. Using a combination of CBFV and arterial blood pressure pulse waveform data, ICP can be estimated through a variety of modeling techniques including regression and machine learning algorithms (28–31). Some of these approaches require a “gold standard” ICP measurement on which to learn the relevant relationship between CBFV and ABP whereas others are based on computational models of the intracranial compliance and resistance (32, 33). However, these models require very careful signal acquisition and alignment, rest on reasonable but simplified assumptions, and ultimately estimate a mean ICP. Therefore, despite the complex signal processing required to generate an ICP estimation, the resulting signal does not retain the information contained within the ICP pulse wave. For the purposes of this review, we will focus on directly measured ICP signals.

### Layer 1: Data Processing

ICP signals are frequently contaminated by noise or artifacts that may hinder peak detection and applications, such as accurate forecasting of elevations in ICP. Therefore, it is essential to understand, filter, and remove noise before implementing any data analytical tools. There are two types of noise for a given signal: high-frequency noise and low-frequency noise. High-frequency noise corrupts ICP recordings whereas low-frequency noise occurs in the same frequency band as important characteristics of the underlying data, and thus requires more nuanced data cleaning processes. ICP signals are non-stationary and non-linear with a frequency spectrum that is not consistent over time. Unlike electromyography (EMG) or electroencephalography (EEG), reference signals are not available in the case of ICP monitoring. Further, magnetoencephalography (MEG), electrocardiography (ECG), or EMG can be considered as the summation of multiple statistically independent components, which assumes that the subcomponents are non-Gaussian signals that are statistically independent from each other (34, 35). The noise components can thus be removed based on thresholding statistical features of components like kurtosis and variance (36). However, this assumption does not hold for the ICP signal (37). These characteristics limit the performance of some popular signal processing methods for artifact removal, such as adaptive filtering or independent component analysis (ICA) (37).

#### Data Processing: Filtering

**Figure 4:**
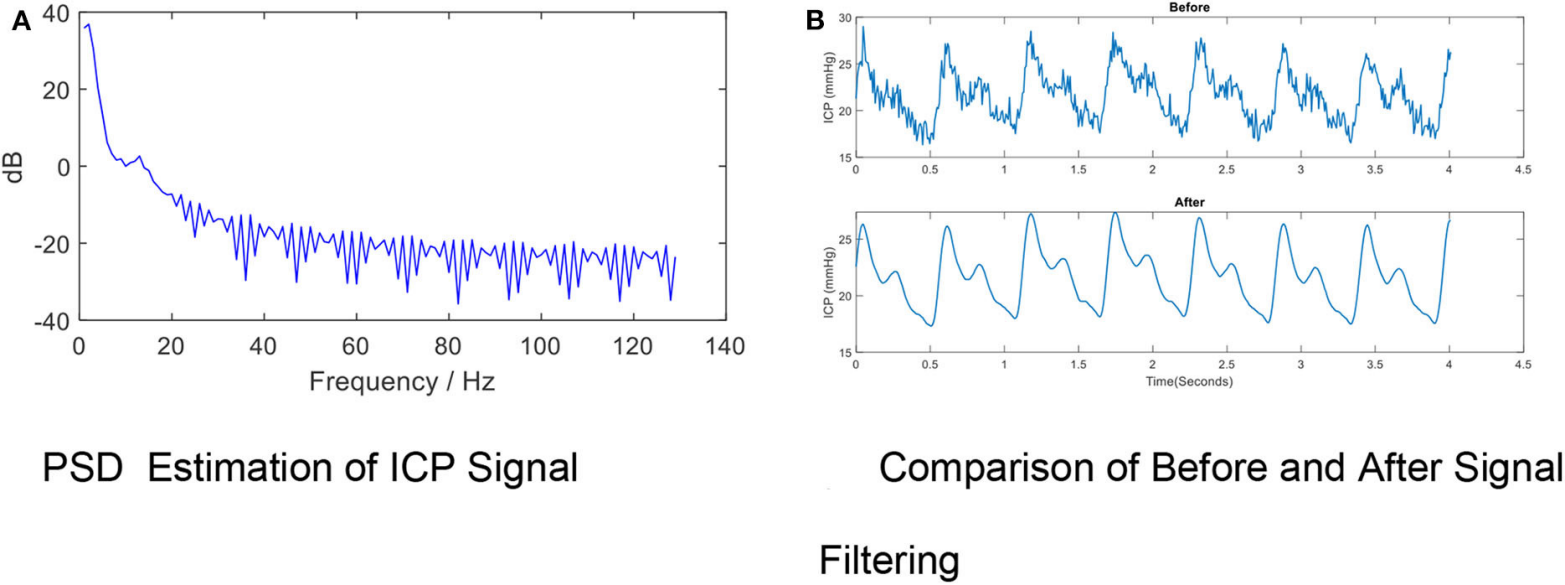
High- frequency Noise Removal for ICP signal. **(A)** PSD estimation of ICP signal. **(B)** Comparison of before and after signal filtering. The high frequency noise can be removed by low-pass filtering. The periodogram is obtained using fast Fourier transform (FFT). In order to conserve the total power, both the positive and negative frequencies multiply by a factor of 2. The zero frequency (DC) equation is $psdx=\;\frac{1}{Fs\times N}\times {\left|{fft\left(x\right)}_{1:\frac{N}{2}+1}\right|}^{2}$, where *psdx* is the estimated power spectral density, *Fs* is the ICP signal sample rate, *N* is the ICP total sample size and *fft* is the FFT function. The PSD results indicate the cut-off frequency rate can be set to 10 Hz in other cases because most of the energy associated with the signal is confined under the threshold of 8 Hz. **(B)** shows the effects of removing high-frequency noise from the ICP signal *via* a low-pass filter (Fs = 128, Fpass = 10 Hz, Fstop = 15 Hz, Apass = 1, Astop = 50, minimum order).

High-frequency noise originates from measurement and amplifier devices, electrical interference, and random quantization noise (3). It is relatively straight-forward to remove these components through traditional filtering methods, such as low-pass filtering. The cut-off frequency rate is typically set at 40 Hz (37–39) in accordance with the Nyquist–Shannon sampling theorem, considering that the biological signal components within the ICP signal cannot reasonably exceed the heart rate. The cut-off frequency rate may be set to 10 Hz in other cases because most of the energy associated with the signal is confined under the threshold of 8 Hz (40, 41), as shown by Figure 4A (40).

A variety of low-pass filters can be designed. The finite impulse response (FIR) filter is recommended over the infinite impulse response filter because a linear phase response is important to preserve the wave-shape of the signal. FIR filters tend to be preferred for fixed-point implementation in ICP signals which are robust to quantization effects. Figure 4B shows the effects of removing high-frequency noise from the ICP signal via a low-pass filter (F_s_= 128, F_pass_ = 10 Hz, F_stop_= 15 Hz, A_pass_ = 1, A_stop_ = 50, minimum order).

#### Data Processing: Artifact Removal

**Figure 5:**
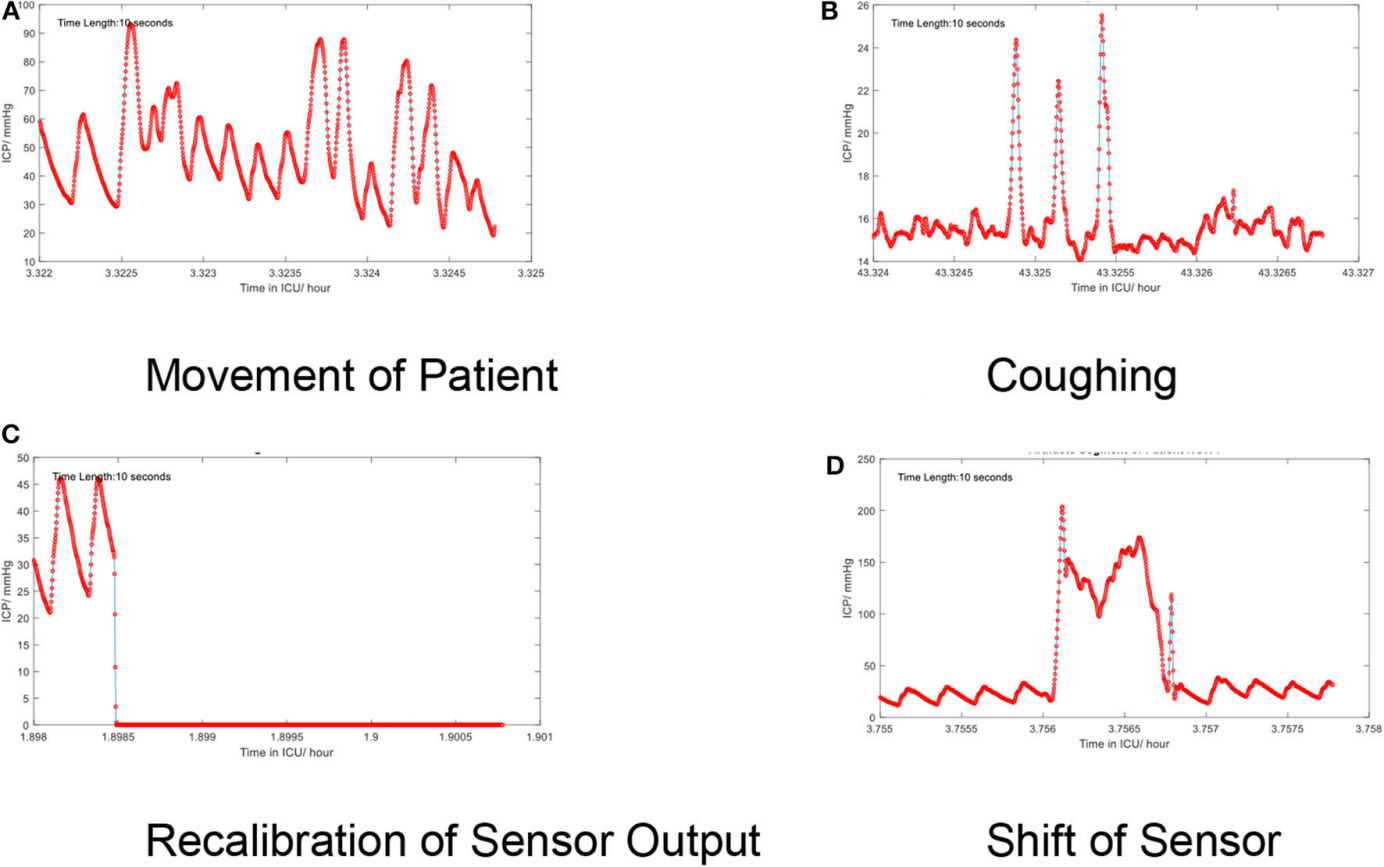
**(A–D)** Artifacts in the time series of the continuous ICP signal. Several low-frequency artifacts are shown.

Low-frequency noise in the ICP signal may be recognized as artifact embedded within the higher frequencies of the signal itself. These artifacts originate from environmental factors which may occur during routine care (e.g., adjustment of the bed angle, movement of the patient, coughing, shifts of the sensors, recalibration of the sensor, or connection errors). The artifacts also depend on types of devices. For example, modern intraparenchymal fiberoptic sensors do not typically require “zeroing,” whereas external ventricular drains may be clamped or require releveling in order to provide accurate measurement. Some examples of artifacts are shown in Figure 5.

#### Artifact Removal: Signal Decomposition Approaches

Signal decomposition techniques such as Empirical Mode Decomposition (EMD), Wavelet Transformation, and Median Filtering are used to recover the underlying structure of the signal of interest.

Many artifacts in the mean ICP time series are visually recognized as tall and sharp “spikes.” EMD is thus employed as a filter to extract variability or changeability of different scales (37). EMD decomposes the mean ICP signals into several Intrinsic Mode Functions (IMFs). An IMF is defined as any function having the same number of zero crossings and extreme values, and also having symmetric envelopes defined by the local maxima and minima, respectively (42). Based on this observation, the first IMF component with large oscillations may be an effective indicator of artifacts. In a study using EMD to remove artifacts across 203.5 h of mean ICP signal, EMD achieved an average of 82% accuracy and F score of 0.848 (37).

The wavelet transform is sensitive to abrupt changes in the mean ICP signal using the Haar basis function. The mean ICP signal is transformed into aggregate energy based on the Haar wavelet and then the first deviation is extracted, which may act as an indicator for artifacts. A median filter is a non-linear digital filter that can be used to extract trend components in ICP signal and the residual component can be regarded as an effective indicator for artifacts (43). Both wavelet and median filters were found to outperform EMD with regard to mean square error (MSE), relative absolute error (RAE), and forecast error (FER) (43). In addition, the median filter method was found to be computationally more efficient, and thus may have more potential for online or real-time application.

#### Artifact Removal: Dominant ICP Pulse Approaches

Dominant ICP pulses refer to the clustering of different morphological characteristics extracted by frameworks such as the Morphological Clustering Analysis of ICP Pulse (MOCAIP) (44). An averaging process is used to obtain an averaged waveform for the largest cluster of ICP pulse waves, referred to as the dominant ICP pulse. One of the benefits of the dominant ICP pulse is the robustness to effects of artifacts due to the averaging process, but simultaneously this has the potential to remove large fragments of potentially useful signal.

A simple threshold-based approach (TB) can be used to recognize artificial ICP pulses if the wave amplitude is between 1.0 and 35.0 mmHg and latency between 0.08 and 0.4 s. Wave amplitude is the pressure difference between the starting diastolic minimum pressure and systolic maximum pressure, while wave latency is the time interval when the pressures change from diastolic minimum pressure to systolic maximum pressure (45).

The dominant pulse peaks could be artifacts if the whole segment consists of noise. Therefore, a non-artifactual ICP pulse recognition approach has been introduced with a template matching (TM) method. A reference library of non-artifactual ICP pulses from multiple patients are constructed. If the dominant pulse correlates with any of the reference pulses with a correlation coefficient greater than a certain threshold, it is recognized as non-artifactual. A self-identification method is also incorporated in order to handle those dominant pulses that are excluded in the reference library. An assumption is made that artifactual dominant pulses are less coherent than non-artifactual ones. Coherence can be defined as the average of the correlation coefficients between pulses compared to the average pulse. This approach was validated by comparing non-artificial pulse recognition with a human labeled ICP pulses and demonstrated a final recognition accuracy of 97.84% (46).

Besides the TM method, an ICP stability algorithm (IS) was developed (45) in which the amplitude *A*_*i*_ and duration *D*_*i*_ of a dominant pulse are compared with the mean amplitude *A*_*m*_ and duration *D*_*m*_ of the past and future three pulses.

$$\begin{array}{l} A_{m}=\;\frac{\overset{-1}{\underset{j=-3}{\sum }}A_{i+j}+\overset{3}{\underset{j=1}{\sum }}A_{i+j}}{6}\\ P_{m}=\;\frac{\overset{-1}{\underset{j=-3}{\sum }}P_{i+j}+\overset{3}{\underset{j=1}{\sum }}P_{i+j}}{6} \end{array}$$

The pulse is recognized as non-artifactual ICP if $\frac{\left|P_{i}-P_{m}\right|}{P_{m}}\leq 15\%$ and $\frac{\left|A_{i}-A_{m}\right|}{A_{m}}\leq \;10\%$.

One recent study leveraged an active learning (AL) framework, further enhancing the identification of non-artifactual ICP signals (45). AL is a semi-supervised learning approach that incorporates a learning model that interactively generates data that is then labeled by experts. It provides an efficient platform for experts that can release them from labeling longer segments of similar samples. The Cohen's Kappa statistic is used to evaluate similarity in following situations: (1) a dataset manually labeled by multiple experts (2) labels classified in AL models which include logistic regression and (3) labels classified by AL models on unlabeled data. The AL based framework exhibited the highest area under the receiver operating curve (AUC) (0.96±0.012), compared to existing threshold based (0.5±0.02), template machine (0.71±0.04), and stability-based (0.51±0.036) methods (45).

#### Data Processing: Signal Segmentation

Signal segmentation is a fundamental tool of signal processing and refers to dividing a signal into epochs prior to further analysis. ICP signal segmentation falls into two categories: (1) frame-by-frame and (2) beat-to-beat. The former is often applied in extracting the mean ICP, usually defined as a 3–8 s moving average of the ICP peak amplitude (10). Frame-by-frame signal segmentation is sensitive to artifacts and outliers thus artifact removal or imputation is required, particularly in order to extract morphological features embedded within the non-artificial pulse. Beat-to-beat segmentation means that the raw ICP signals can be converted into dominant ICP pulse waveforms with the assistance of other signals for example ECG, plethysmography, or arterial blood pressure (ABP) waveforms.

Hu et al. leveraged the established technique of ECG QRS detection to first identify each ECG beat (44, 46). The ICP latency was then defined as the time interval between the peak of the QRS of the ECG and the onset point of ICP pulse. The average ICP latency was reported as 72.6 ± 19.5 ms. By combining the ECG QRS-based ICP latency extraction algorithm and an adaptive peak detection algorithm, the ICP pulse detection algorithm reached a baseline sensitivity of 0.97 and an accuracy of 0.88.

Others have used ABP pulse-wave signals because they also carry information about the real-time variation of the pulsatile waveform within the cardiac cycle (47). However, this approach may be limited as the ABP signal is frequently contaminated by artifacts requiring independent detection and removal as part of its preprocessing.

### Layer 2: Information From ICP Signal Time-Series

The mean ICP refers to the average of the peak amplitude across specific windows of time, as described above. The mean ICP is the value typically used clinically to guide therapeutic interventions to lower the mean ICP, such as hyperventilation or mannitol administration. However, the mean ICP is only one feature that can be generated from the ICP signal over time. The information contained within the ICP pulse signal can be generally divided into four categories in complex systems terminology (48): time domain, frequency domain, scale invariant features, and entropy. In contrast to morphological analysis, which focuses on the features of individual ICP waveforms, ICP signal time-series information is extracted across pre-specified time-windows.

#### Information: Time Domain Features

Time domain information refers to variations in the amplitude of a signal along with time. The clinical use of “mean ICP” is an example of a time domain feature of the ICP pulse signal over time. Other forms of time domain analysis were popular in the 1970's, such as the peak-to-peak pulse pressure amplitude (49). More recently, time domain information has been used to extract *multiple* features, including: statistical summary features (maximum value, average, and standard deviation), covariance coefficients, similar to what is used in EEG analysis, and higher-order time domain features such as coefficients of absolute difference, autocorrelation coefficients, and instant ICP values (50). The power of using time-domain features is that they exist in an intuitive feature space and can be used to develop real-time alarms. For instance, after transforming the ICP to a time domain feature space, hierarchical Gaussian mixture models (hGMMs) can be adopted to develop novel alarm functions based on posterior probabilities (50).

The ICP pressure burden is a feature in the time domain which combines the clinically-standard mean peak ICP feature and time information. It may be defined as the mean daily duration of time that the mean ICP is recorded above a pre-specified threshold [e.g., 25 mmHg (51)]. ICP pressure burden can also be calculated as “pressure times time dose” (mmHg·h) over a pre-specified threshold (52). Alternatively, geometric approaches can be used to define pressure burden as a time-weighted elevation in ICP, such as the trapezoidal method (53). In this method, the mean ICP is calculated by approximating the integral of the curve of the ICP trend line for each patient. Then, the AUC is calculated by the trapezoid rule: $0.5\times \left(T_{i+1}-T_{i}\right)\left(ICP_{i}^{+}+ICP_{i+1}^{+}\right)$, where *T*_*i*+1_−*T*_*i*_ represent the time interval between the *i*th and *i*+1 time window and the $ICP_{i}^{+}$ represents the ICP values that exceeded the threshold, in this case 20 mmHg. The AUC value is then divided by the duration of the whole monitoring time (53). Count methods may be used with higher-resolution data and in one study, minute-to-minute mean ICP was visualized using a map of intensity vs. duration and color-coded based on correlations with group-based outcome. A time-dependent relationship was observed wherein longer durations of time spent at lower thresholds of mean ICP and shorter durations of time spent at higher thresholds of mean ICP increased the odds of poorer outcomes (54).

Intracranial pressure variability (IPV) can be extracted by the successive variation (SV) method. SV is the average difference in the mean ICP between two successive parameters. $SV=\;\sqrt{\frac{1}{\left(n-1\right)}\overset{n-1}{\underset{i=1}{\sum }}{\left(X_{i+1}-X_{i}\right)}^{2}}$, where *X*_*i*_ is the ICP value at *i* time stamp and n is the number of time points. SV1 can be calculated from using each hour's mean ICP value (*X*_1_, *X*_2_, *X*_3_…, *X*_*n*_); SV2 is defined as the average of consecutive pairs of ICP data ($X_{21},\;X_{22},X_{23}\ldots ,X_{2\frac{n}{2}})$ where $X_{21}=\frac{X_{1}+X_{2}}{2}$. ICP-SV2 was associated with 30-day functional outcome in one study (55).

Time domain features are also useful in conjunction with time-windowing in order to create moving average correlation coefficients between ICP signal features or between the ICP signal and other physiologic parameters within the same time window. Cerebrospinal compensatory reserve (RAP, which stands for the correlation *R* between the pulse amplitude *A* and the pulse pressure, or ICP, *P*) is calculated as moving average correlation coefficient between the ICP pulse amplitude, defined as the difference between the peak amplitude of the ICP signal and the trough of the ICP signal and the mean ICP averaged over a 10 s window (56). The RAP serves as an index of compensatory reserve and predictor of neurological deterioration (57). A RAP value of 0 indicates that as the pulse amplitude increases, the ICP does not—an indication of compensatory reserve, as a change in volume (in this case inferred by the pulse amplitude) does not result in substantial changes in intracranial pressure. In contrast, poor compensatory reserve occurs when the pulse amplitude increases alongside the mean ICP, resulting in a RAP closer to one. At the extreme end of the pressure-volume relationship, the RAP becomes −1 as there is loss of autoregulatory capacity and vessels become passive to external compression from elevated ICP (58). As useful as this may be, the RAP does have limitations based on its use of the mean ICP which may be prone to errors (as described above) and the pulse amplitude alone may be a more reliable marker of critical changes in cerebral blood volume and therefore pressure (59).

Pressure reactivity index (PRx) is another form of correlation analysis between time domain features and has been particularly well-studied. The PRx is calculated as a moving average correlation coefficient between the 10-s mean of the arterial blood pressure (ABP) signal and the mean ICP signal over a 5-min time window (60). PRx quantifies cerebrovascular reactivity and approximate global cerebral autoregulatory reserve by observing the responses of each signal to slow spontaneous changes. PRx values that are negative or close to zero indicate preserved autoregulation, whereas PRx values closer to one indicate impaired autoregulation (61). The PRx can subsequently be plotted against the perfusion pressure, which demonstrates a characteristic *U*-shaped curve in ~60% of patients with TBI undergoing ICP monitoring (62). The nadir of the curve indicates the CPP at which the PRx, or autoregulatory capacity, is optimized. This *optimum CPP* varies patient-to-patient but in a retrospective study of 327 patients, the average optimum CPP was 75 mmHg, higher in comparison to the Brain Trauma Foundation Guidelines that suggest an empiric CPP 60–70 mmHg. Further, CPP values that fell outside the optimum were associated with higher mortality and disability (63).

#### Information: Frequency Domain Features

Frequency domain features are usually calculated using spectral or wavelet analyses. Initially, the ICP signal is projected from time domain to frequency domain using algorithms such as the fast Fourier transform (FFT). The discrete wavelet transformation (DWT), similarly, divides the waveform into component sinusoidal waves based on different frequency bands (64). Using DWT, the high-frequency centroid of the ICP signal has been defined as the power-weighted average frequency within the 4–15 Hz band of the ICP power density spectrum. A rapid increase in the high-frequency centroid frequency was found to be associated with deterioration caused by expanding intracranial hematomas (65). The frequency domain-based amplitude (AMP) can be extracted using spectral analysis and was shown to be an effective metric wherein higher AMP was correlated with decreased brain compliance (66, 67). However, frequency domain-based analysis may lose information when converting the signal from time domain to the frequency domain. Holm and Eide showed that the frequency domain method could underestimate pulse amplitude if there is heart rate variability or high harmonic distortion. Thus, the time domain method is superior to the frequency domain method with respect to preserving the pulse amplitude (68).

#### Information Scale (Fractal) Invariant Features

Scale (fractal) invariance is a feature of objects that do not change if scales of length, energy or other variables are multiplied by a common factor, and thus represent universality. Detrended Fluctuation Analysis (DFA) describes the second-order statistical properties of signals that may reflect long memory processes, long-range correlations, fractal scaling, and evolving intrinsic non-stationarities (69). One study explored the association of two DFA-based ICP coefficients, namely the scaling exponent and the intercept, with 6-month functional outcome, while controlling for the initial neurological exam. They found that lower DFA intercept values and higher scaling exponent values were significantly associated with unfavorable outcome in a sample of 147 moderate-to-severely injured TBI patients (70). The scaling exponent, similarly, has been shown to significantly increase during periods of elevated intracranial pressure in a study of 30 patients with severe TBI (71). The scaling exponential can also be combined with measures of entropy to better reflect the complexity of the ICP signal (69).

#### Information: Entropy Features

Entropy is a concept that is used to quantify randomness, unpredictability, or irregularity within a system. Approximate entropy (ApEn) is the most commonly used entropy calculation and serves as a benchmark for other entropies. ApEn is a family of parameters and statistics that can quantify regularity in data without any prior knowledge about the system generating them (72). In one study, the mean of the ApEn of the ICP signal was reduced during periods of elevated ICP (>25 mmHg) for 5 min or less (73). Sample entropy (SampEn) was introduced as an advancement over ApEn specifically for physiological time-series signals (74). SampEn addresses the problems of eliminating self-match compared with ApEn, which requires massive data and larger time windows to avoid an overestimation of regularity. While SampEn has not been used for ICP signals, a modification termed Multiscale Entropy (MSE) has been used (75). MSE attenuates the stationarity assumption of SampEn by taking into account more than one temporal scale in its calculation and as such it is more robust than classical entropies in describing the complexity of signals. For instance, ApEn and SampEn tend to falsely judge white noise as having high complexity due to its randomness, whereas MSE recognizes the relatively low complexity of the randomness. Therefore, MSE may be preferable to single-scale entropy-based analyses for signals such as ICP (76). The MSE of the ICP signal was found in one study to be superior in predicting functional neurological outcome compared with other measures of entropy. In this study of 290 patients with a range of TBI, reductions in the MSE of the mean ICP was associated with poor outcome (75).

Wavelet entropy is a relatively novel entropy based on energy distribution in wavelet sub-bands (77, 78). The traditional spectrum estimation would be easily affected by noise and artifact; therefore, segmenting and windowing procedures have been added to the calculation of wavelet entropy and relative Wavelet Entropy (rWEn) was introduced to measure the dissimilarity of between two ICP signals (78). The advantages and limitation of these four entropies are illustrated in Table 2.

**Table 2:** Summary of approximate entropy, sample entropy, wavelet entropy and multiscale entropy.

**Entropy**	**Interpretation**	**Advantages**	**Limitations**
Approximate Entropy (ApEn)	The larger the ApEn, the less the predictability or the higher the randomness	1. ApEn correlates with hidden and subclinical changes often undetected by other classical time series analysis (moment statistics, spectral analysis, and correlation analysis) 2. ApEn can assess subtle disruption, typically preceding change in signal mean and standard deviation	1. The higher entropy value only indicates an increase in the degree of randomness rather than complexity 2. The calculation usually require very long data sets and a bias may exist leading to overestimation of the time series regularity
Sample Entropy (SampEn)	The larger the SampEn, the less the predictability or the higher the randomness The larger value of SamEn, the less self-similarity	1. Simpler than ApEn 2. Largely independent of record length and thus consistency 3. Less biased than ApEn since it eliminates self-matches	1. The estimation of SampEn critically depends on the selection of the parameters' sequence length 2. The stationarity assumption is invalid for prolonged time periods
1. Wavelet Entropy (WEn) 2. Relative Wavelet Entropy (rWEn)	The larger the wavelet entropy, the less the predictability or the higher the randomness	1. Wavelet entropy has similar performance with ApEn 2. Inherits the high computational efficiency of wavelet decomposition 3. rWEn could be further used to measure dissimilarity between two time series signals	1. The higher entropy value only indicates an increase in the degree of randomness rather than complexity 2. Parameter selection in wavelet decomposition could cause bias in clinical practice
Multiscale Entropies (MSE)	The larger the multiscale entropy, the increase degree of complexity	1. Characterize complexity in signal better than other entropies 2. Multiscale entropy can attenuate the effect of the stationarity assumption in the underlying distribution of signals	Multiscale entropy requires substantially more samples than single scale sample entropy

### Layer 2: Information From ICP Morphology

Changes in the configuration of the three ICP pulse peaks may be a relevant indicator of cerebrovascular pathophysiology. For example, in clinical practice the ICP pulse is often reviewed at bedside and a dominant P2 wave is used to infer abnormal cerebral compliance (70). The *quantification* of morphological features is challenging, as discussed previously and, to date, the clinical usefulness of quantified ICP morphology has been restricted by these limitations.

As introduced above, the Morphological Clustering and Analysis of ICP Pulse (MOCAIP) framework was developed to allow for systematic quantification of the ICP waveform based on its morphology and to understand its application using continuous ICP waveforms (46, 79, 80). The MOCIAP was first proposed to identify non-artificial dominant ICP and optimally designate three sub-peaks in an ICP pulse (46). An automated and robust system was subsequently developed to extract the morphological features of the real-time ICP waveform. These features allow a comprehensive quantitative characterization of the ICP waveform including amplitude, time intervals between sub-peaks, curvature, slope, and time decay constants. This approach was validated by manually labeled datasets with an accuracy of 90.17, 87.56, and 86.53% for each of three sub-peaks P1, P2, and P3, respectively. A total of 24 MOCAIP metrics or features have been developed and validated (80).

Although high accuracy is achieved by using dominant ICP clustering, morphological clustering using only ECG signals has the potential to lose the characteristics of individual pulse signal peaks in relation to their arterial inputs. By introducing simultaneous ABP signals, ICP pulse morphologies can be delineated by landmarks including peaks, troughs, and flats. Then, rule-based and modified *K*-means clustering algorithms can be used for peak clustering. This algorithm successfully identified the three distinguishing peaks of the ICP with satisfactory accuracy: 95.3, 87.8, and 87.5% for P1, P2, and P3 (47).

Morphological analysis of ICP has traditionally been limited in performance due to the appearance of artifacts. The artifact and noise from the ICP signal may introduce unwanted error and ultimately create unreliable data for clinical monitoring and management. Current morphological feature extraction techniques used for research purposes require manual artifact removal and access to an ICP morphological reference library, although newly developed AL frameworks (45) and similar data analytic frameworks to what we propose here may mitigate these limitations. Though some have suggested that the ICP artifacts account for <10% of total data (47), ICP artifact detection may be time-prohibitive or create other barriers to implementing these algorithms online.

### Layer 3: Modeling and Application of ICP Signal Processing Techniques

There are two primary objectives that have been described for the applied use of ICP pulse signals in neuromonitoring. The short-term term goal is to detect or predict elevated ICP and other secondary brain injury patterns. The long-term goal is to link novel ICP features with clinical outcomes and to use this information to create ICP signal-based alarms based on these predictions. Future goals will need to focus on the coupling between ICP features and other physiological parameters, but these studies to date have been largely exploratory and fall outside the scope of the current discussion.

Intracranial hypertension, or secondary brain injury, prediction (IHP) uses features called *precursors* that always occur several minutes ahead of the onset of elevated ICP. In contrast, features used to detect critical intracranial hypertension (IHD) are within the period of time in which the mean ICP is elevated. Both IHP and IHD are formulated as classification problems, although IHP is more challenging since the precursors are not readily distinguished. Long-term objectives instead focus on data modeling. Long-term outcome evaluation (LOE) mostly leverages statistical modeling such as regression, which deals with the relationships between variables to infer an outcome. Long-term outcome prediction (LOP) employs machine learning algorithms that can learn from test data in order to predict outcome without a rule-based approach. The outputs from LOE are the relationship parameters (e.g., significance levels between predictor features and long-term outcomes; LOP emphasizes the *performance* of the predictive models). The applications and modeling of ICP pulse signals are summarized in Table 3.

**Table 3:** Summary of modeling and application in ICP signal.

**References**	**Application**	**Features or information**	**Modeling approach**	**Performance or delivery**
Quachtran et al. (81)	IHD	CNN based features	Regression; CNN, CNN + Autoencoder ^*^	ACC: 92.05%
Scalzo et al. (82), Scalzo and Hu (39)	IHD	MOCAIP Metrics improved by CDF; Trending features	Threshold-based; SR-DA, SVM, SR-KDA^*^	AUC: 85.9%; Reduce FPR by 31%
Soehle et al. (71)	IHD	Complexity (scaling exponent, SampEn, multiscale entropy)	Statistical analysis	scaling exponent (*p* < 0.001), SampEn (*p* = 0.004), MSE (*p* < 0.05)
Scalzo et al. (41)	IHP	MOCAIP Metrics with backward sequential feature selection	MLR; Adaboost; Extra-Tree^*^;	AUC: 96%, SPE: 98%, SEN: 93% (1 min prior to the elevation)
Xiao et al. (80)	IHP	Optimal MOCAIP metrics found by DE algorithms	Regularized quadratic discriminator	SPE: 99%, SEN: 37% (5 min prior to the elevation)
Hamilton et al. (83)	IHP	Top 10 MOCAIP metrics found by PSO	A quadratic classifier (QDC)	ACC: 77%, SEN: 90%, SPE: 75% (5 min prior to the elevation)
Hornero et al. (73)	IHD	ApEn	Statistical analysis: non-parametric bootstrap hypothesis testing	*p* < 0.01 (IH group vs. recovering group)
Teplan et al. (50)	LOP	7 Time domain features	hGMMs, ROC analysis	YI = 0.33, SPE: 85%, SEN: 48%
Pimentel et al. (84)	LOP	GPs based dynamic features, PRx-based statistical features	GPs-based, PRx-based, Combined Model^*^	ACC: 74%, SPE: 65%, SEN: 83%, AUC: 76%
Güiza et al. (54)	LOE	Insult Intensity (I), Insult duration (D), LAx	Multivariate logistic regression models and color-coded plot	ICP- time burden Visualization,
Howells et al. (8)	LOE	Pressure reactivity indices in various frequency band	Statistical analysis: correlation, Spearman's R, two-tailed Wilcoxon matched pairs test	ρ = −0.46 (correlation with GOSe)
Lazaridis et al. (85)	LOE	PRx, Cumulative area under the curve above threshold	Logistic regression models,	AUC: 0.77, 95% CI 0.70–0.83
Lu et al. (75)	LOE and LOP	Multiscale entropy	Statistical analysis: ANOVA, Logistic regression	Favorable (*F* = 28.7, *p* < 0.0001), Unfavorable (*F* = 17.21, *p* < 0.0001) ACC: 82%, SPE: 94%, SEN: 50%

IHD, Intracranial Hypertension Detection; IHP, Intracranial Hypertension Prediction; LOE, Long-term Outcome Evaluation; LOP, Long-term Outcome Prediction; ACC, Accuracy; SPE, Specificity; SEN, Sensitivity; AUC, Area Under receiver operating characteristic Curve; CNN, Convolutional Neural Networks; DE, differential evolution; hGMMs, hierarchical Gaussian mixture models; YI, Youden index; GPs, Gaussian processes; Lax, low-frequency autoregulation index; GOSe, extended Glasgow Out- come Scale; ANOVA, Analysis of Variance; ApEn, Approximate Entropy; PSO, Particle Swarm Optimization; SR-DA, Spectral regression- discriminant analysis; SR-KDA, Spectral Regression-Kernel Spectral Regression.

* *is the algorithm with best performance*.

#### Application: Intracranial Hypertension Detection or Prediction

Intracranial Hypertension (IH) is defined by elevations in ICP that are thought to potentially compromise blood flow and therefore create secondary brain injury. The recommended threshold for the treatment of ICP after severe TBI is above 22 mmHg, although individual thresholds above which brain tissue and blood flow are compromised are unknown (2). Therapeutic interventions are typically targeted once elevations in ICP occur for a pre-specified amount of time (41). Since the pressure-time ICP burden appears to correlate with outcome, IH detection and prediction can potentially lead to earlier intervention.

Formulating a detection or prediction problem in IH relies on the definition of selected data, namely pre-IH episodes, IH episodes or control episodes. One study defined IH episodes as mean ICP values of more than 20 mmHg over a period of at least 5 min (80, 83), while another used ICP values more than 30 mmHg lasting at least 10 min (86). Pre-IH episodes are usually selected as 5, 10, 15, and 20 min prior to onset of ICP elevation (80, 83). Control episodes are selected either from segments at least 1 h prior to ICP elevation from patients with at least one episode of IH, or segments from patients without a single episode of IH (80). If only IH and control episodes are compared, the problem would be IH detection (81, 87), whereas IH prediction compares the pre-IH episodes and IH episodes.

**Figure 6:**
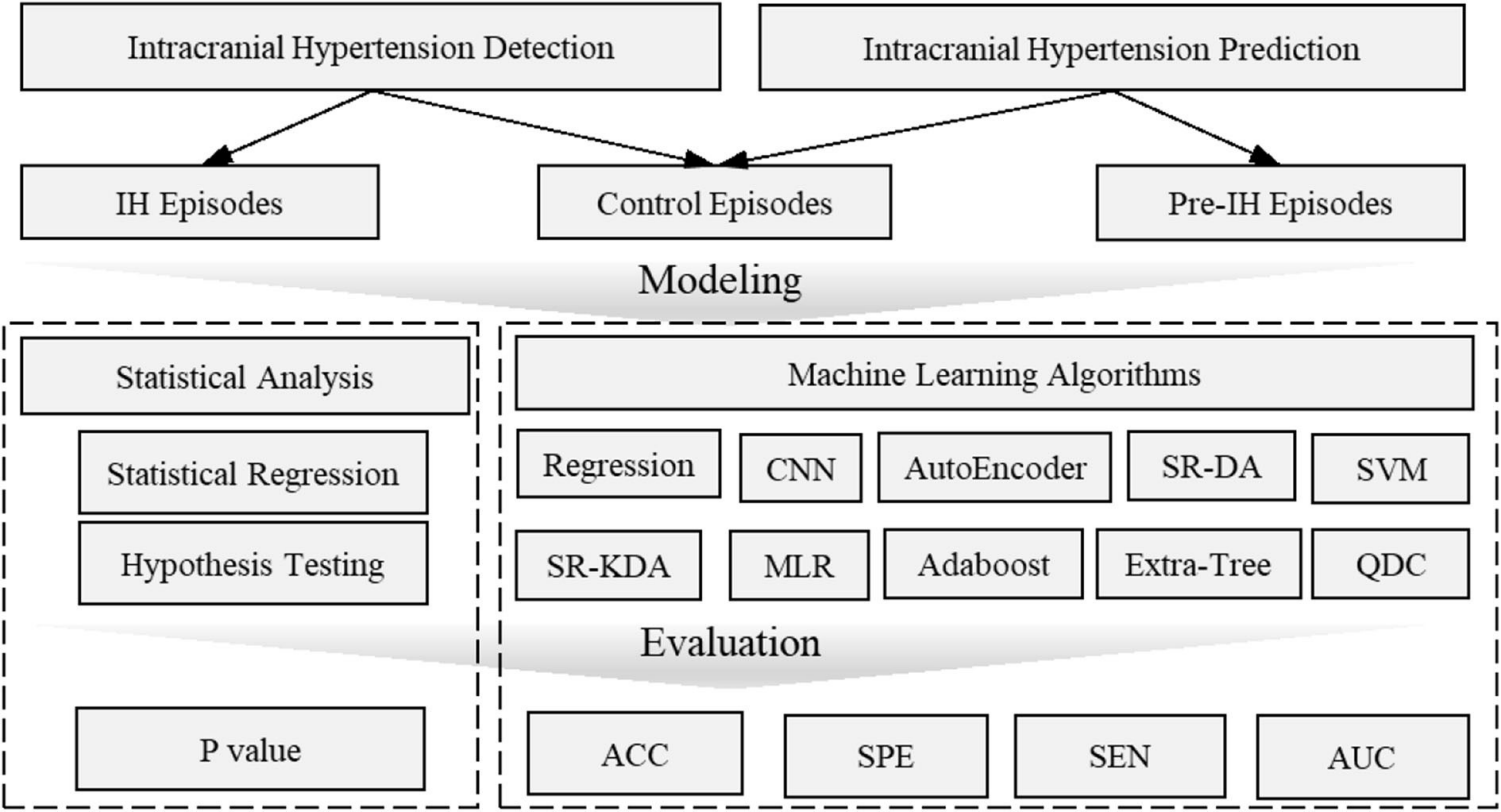
Overview of intracranial hypertension detection and prediction modeling. Intracranial hypertension detection uses intracranial hypertension episodes and control episodes while intracranial prediction uses pre-hypertension episodes and control episodes. The statistical analysis is usually evaluated by *p-*value which is concerned with the significant difference of individual features between pre-intracranial hypertension/intracranial hypertension and control groups. Machine learning models are often evaluated by accuracy, AUC values etc. to demonstrate the predictive power to distinguish pre-intracranial hypertension/intracranial hypertension groups from other control groups. ACC, Accuracy; AUC, Area Under receiver operating characteristic Curve; CNN, Convolutional Neural Networks; MLR, Multivariate linear regression; SEN, Sensitivity; SR-DA, Spectral Regression- Discriminant Analysis; SVM, Support Vector Machine; SPE, Specific; SR-KDA, Spectral Regression-Kernel Spectral Regression; QD, Quadratic Classifier.

From a modeling perspective, the two approaches, namely statistical analysis vs. machine learning and other artificial intelligence algorithms, can be distinguished based on the final goal. Machine learning models often involve many more explanatory variables or features than statistical models, while statistical analysis is usually concerned with the *significance* of individual features. Machine learning models focus on optimizing predictive power using combinations of a large number of features. Predictive power is then evaluated by accuracy, sensitivity, and AUC (area under receiver operating characteristic curve) through a comparison of actual vs. predicted labels. The labels are usually annotated by experts or known based on clinical course or outcome. The significance of individual features in a typical statistical model is usually evaluated by *p*-values or the confidence intervals surrounding model coefficients and is associated with explanation, not prediction. Hypothesis tests are used to test the validity of a claim about a population. For instance, the null hypothesis might be that a feature (e.g., complexity) is not different between IH episodes and control episodes. A small *p*-value (typically ≤ 0.05) indicates strong evidence against the null hypothesis; the null hypothesis is rejected and a conclusion can be reached that there is a difference in complexity between IH and control groups. An overview of this distinction is shown in Figure 6.

To detect IH episodes, several models have demonstrated the capability of features to detect the difference between IH groups and control groups. WEn analysis has shown that episodes of IH have more focused energy in the low wavelet frequency band (0–3.1 Hz) than control episodes (77). An increase in ApEn between IH episodes and control episodes was determined to be statistically significant *p* < 0.01 (73). SampEn was significantly reduced during the IH period (ICP = 31.7 ± 7.8 mmHg, SampEn = 1.45 ± 0.46, mean ±*SD*) compared to control episodes (ICP = 15.7 ± 3.2 mmHg, SampEn = 1.45 ± 0.46; *p* = 0.004). The scaling exponent α derived from detrended fluctuation analysis significantly increased (α = 1.02±0.22) during IH episodes compared to control episodes with *p* < 0.001 (71).

The development of machine learning algorithms has allowed models to incorporate more features in order to enhance the predictive power of IH detection. The MOCAIP algorithm was leveraged to extract 24 morphological features for individual ICP episodes and an optimal subset of these morphological features was then determined using a global optimization algorithm. Four machine learning algorithms, spectral regression (SR), discriminant analysis (DA), kernel spectral regression (SR-KDA), and support vector machines (SVM) were then introduced to reduce the false alarm rate generated from traditional threshold-based technique. The SR-KDA achieved the best performance with AUC 85.9% and reduced the false alarm rate by 27% compared to a conventional threshold-based approach (82). A semi-supervised learning-based framework using SR-KDA and SVM on unlabeled samples led to FPR reductions to 9% (supervised) and 27% (semi-supervised) for SR-KDA, and to 3% (supervised) and 16% (semi-supervised) for SVM (39). More recently, deep learning has been utilized to extract features that are characteristic of IH episodes. A three-layer Convolutional Neural Network (CNN) was trained on MOCAIP data and tested with a 3-fold cross-validation; its accuracy reached 87.19% (82). An autoencoder was used to reconstruct the features for pre-training enhancement and increased the accuracy further to 92.05% (81).

IH prediction has been largely focused on the range of morphological features (e.g., MOCAIP). A quadratic classifier (QDC) model was introduced with Particle Swarm Optimization (PSO) for locating the optimal combination of feature subsets. The top 10 MOCAIP metrics achieved an adequate accuracy (0.77), specificity (0.75), and sensitivity (0.90) to predict IH 5 min *prior* to an ICP elevation (83). The MOCAIP features were further selected by a differential evolution algorithm and control episodes vs. pre-IH episodes were classified by a regularized quadratic discriminator. The performance was improved to a specificity of 0.99 and sensitivity of 0.37 5 min prior to an ICP elevation. Up to 20 min prior to ICP elevation, specificity was retained, although the sensitivity was reduced to 21% (80). Further improving this algorithm, backward sequential feature selection was used followed by three classification algorithms: multiple linear regression (MLR), Adaboost classifier and the Extra-Tree algorithm. The Extra-Tree algorithm achieved the best performance with 0.96 AUC, 0.98 specificity, and 0.93 sensitivity 1 min prior to ICP elevation, and 0.85 AUC, 0.99 specificity, and 0.70 sensitivity up to 9 min prior to onset of elevated ICP (41).

**Figure 7:**
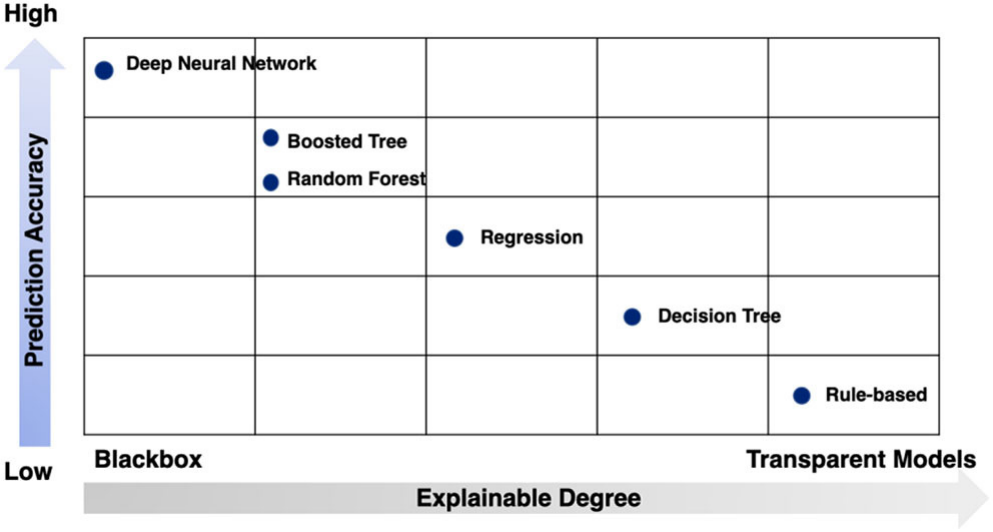
Strengths and limitations of modeling techniques by prediction accuracy and explainable degree.

While no signal method is optimal, the strengths and limitations of these techniques can be conceptualized in two dimensions: prediction accuracy and explainable degree (Figure 7).

#### Application: Long-Term Outcome Evaluation or Prediction

As described, statistical approaches are usually used to model the relationship between ICP signal characteristics and long-term outcome of TBI patients, often measured based on ordinal scales that describe functional outcome, such as the Glasgow Outcome Scale score (GOS) or the Glasgow Outcome Scale Score Extended (GOS-E). An increased PRx, signifying impaired cerebrovascular pressure reactivity, has been shown to be associated with poor GOS in patients after TBI (88, 89) and GOS has been correlated with PRx averaged both over the whole monitoring period (*p* < 0.002) or within the first 24 h (*p* < 0.0001) (89). The MSE of the ICP time-series achieved statistical significance stratifying patients into dichotomous outcomes: death/survival and unfavorable/ favorable (*F* = 28.7;*p* < 0.0001 and *F* = 17.21;*p* < 0.0001). A higher *F*-value indicates better differentiation of a variable across outcome strata. Interestingly, MSE was a more significant factor (*p* < 0.0001 for mortality; *p* < 0.0001 for favorable outcome) than PRx (*p* = 0.23 for mortality; *p* = 0.31 for favorable outcome) (75). The ICP pressure-time dose (mm Hg∙h) showed significant correlation with discharge GOS score (favorable/ unfavorable) with *p* = 0.008 / *p* = 0.038 (52). Pressure burden (53) has also been associated with a composite endpoint of worse functional and neuropsychological outcome.

For logistic regression, the GOS score is often converted to a binary response variable in order to simplify the classification problem between favorable outcome (GOS 4 and 5) and unfavorable outcome (GOS 1, 2, or 3). Using multiple features to assess universal thresholds associated with GOS using logistic regression with a 5-fold cross validation, one study found that three ICP thresholds could be defined: PRx > 0.2, ICP > 20 mmHg, and ICP > 25 mmHg. The PRx threshold had the highest performance to predict the GOS (AUC 0.81, 95% CI 0.74–0.87) over ICP > 20 mmHg (AUC 0.75, 95% CI 0.68–0.81) and ICP > 25 mmHg (AUC 0.77, 95% CI 0.70–0.83) (85). Others have fit logistic regression models to the features extracted from Gaussian process-based models and PRx to predict mortality with model performance of 0.74 accuracy, 0.65 specificity, and 0.83 sensitivity, although this modeling was limited in its clinical utility (84). Of note, most studies do not consider the effects of clinical decisions to withdraw life-sustaining therapies, which may result in differential classification bias.

Multivariate regression using clinical predictors has also been used to strengthen long-term outcome predictive models. Lu et al. used age, GCS (Glasgow Coma Scale), PRx and MSE predictors to forecast the long-term outcome for patients. Using a binary response variable, favorable/unfavorable outcome, the averaged accuaracy, sensitivity, and specificity were 0.82 ± 0.07, 0.50 ± 0.19 and 0.94 ± 0.06 (75). The use of ICP features to predict outcome should be only evaluated in conjunction with clinical parameters, as it is critical to disentangle the independent impact of ICP from the effects of injury severity on the development of elevated ICP and its treatment. The use of multiple physiologic data points (such as simultaneously recorded brain tissue oxygen or regional cerebral blood flow) may provide additional insight.

## Conclusions and Future Work

ICP measurement and monitoring in patients with severe brain injuries is a cornerstone of intensive care and while there is debate about the utility of ICP measurement, it is clear that further research is needed in order to better understand the information contained within the ICP signal and its clinical importance. In this review, we propose an instructive framework for data mining and application of ICP waveform signals. The data processing layer serves to provide ICP signal cleaning for analysis. The information layer generates features to extract discriminative or relative information. Finally, the application layer can model and map the features and information to outcome measures such as intracranial hypertension or long-term functional outcome.

Signal processing and Data science in neurocritical care is rapidly progressing. Over the past 10–15 years, ICP-based autoregulatory indices have been developed, automated ICP morphology extraction has been refined, and predictive models have been developed for clinically-relevant outcomes. However, the use of ICP is limited by several surmountable barriers, namely: (1) real-time or online evaluation and forecasting has not been deployed in clinical ICP monitoring and management; (2) the information contained within frame-by-frame segmented ICP signals need to be further characterized and clinically correlated; (3) specific morphological features need to be prioritized to maximize model fitting and allow for online clinical use; (4) artifact detection, removal and imputation needs to be standardized to allow for the systematic study of ICP waveforms, to promote consistent online monitoring, and for robust machine learning algorithm development; and (5) model development and algorithm construction need to be refined using open-source platforms with robust versioning and regular reevaluation.

ICP monitoring as a system can benefit from advancements in computer science and engineering. The development of Internet of Thing (IoT) (90), big data techniques (91, 92), and CPS (Cyber-Physical Systems) (93) promise to transform our ability to monitor the brain in real time after injury.

## Author Contributions

HD provided the literature review, concept, and manuscript draft. XJ provided critical appraisal and revisions to the manuscript and figures. LP contributed to the concept of the paper and critical revisions. JL contributed to developing the concept of the paper. BF was responsible for guiding manuscript development, contributed to the concept, and critical revisions to the manuscript. All authors contributed to the article and approved the submitted version.

## Conflict of Interest

The authors declare that the research was conducted in the absence of any commercial or financial relationships that could be construed as a potential conflict of interest.

## References

[1] Faul M Xu L Wald MM Coronado V Dellinger AM Traumatic brain injury in the United States: national estimates of prevalence and incidence, 2002–2006. Injury Prev. (2010) 16(Suppl.1):A268. 10.1136/ip.2010.029215.951

[2] Carney N Totten AM O'Reilly C Ullman JS Hawryluk GWJ Bell MJ Guidelines for the management of severe traumatic brain injury, fourth edition. Neurosurgery. (2016) 1:1432 10.1227/NEU.0000000000001432 27654000

[3] Calisto AAAA Galeano M Serrano S Calisto AAAA Azzerboni B. A new approach for investigating intracranial pressure signal: filtering and morphological features extraction from continuous recording. IEEE Trans Biomed Eng. (2013) 60:830–7. 10.1109/TBME.2012.2191550 22453602

[4] Cushing H The Third Circulation in Studies in Intracranial Physiology and Surgery. London: Oxford University Press (1926).

[5] Wilson MH. Monro-Kellie 2.0: the dynamic vascular and venous pathophysiological components of intracranial pressure. J Cereb Blood Flow Metab. (2016) 36:1338–50. 10.1177/0271678X16648711 27174995 PMC4971608

[6] Davson H Hollingsworth G Segal MB. The mechanism of drainage of the cerebrospinal fluid. Brain. (1970) 93:665–78. 10.1093/brain/93.4.665 5490270

[7] Adolph RJ Fukusumi HIROO Fowler NO. Origin of cerebrospinal fluid pulsations. Am J Physiol Legacy Content. (1967) 212:840–6. 10.1152/ajplegacy.1967.212.4.840 6024448

[8] Howells T Johnson U McKelvey T Enblad P. An optimal frequency range for assessing the pressure reactivity index in patients with traumatic brain injury. J Clin Monitor Comp. (2014) 29:97–105. 10.1007/s10877-014-9573-7 24664812

[9] Harary M Dolmans RGF Gormley WB. Intracranial pressure monitoring—review and avenues for development. Sensors. (2018) 18:1–15. 10.3390/s18020465 29401746 PMC5855101

[10] Ellis T McNames J Aboy M. Pulse morphology visualization and analysis with applications in cardiovascular pressure signals. IEEE Trans Biomed Eng. (2007) 54:1552–9. 10.1109/TBME.2007.892918 17867347

[11] Lundberg N Troupp H Lorin H. Continuous recording of the ventricular-fluid pressure in patients with severe acute traumatic brain injury: a preliminary report. J Neurosurg. (1965) 22:581–90. 10.3171/jns.1965.22.6.0581 5832775

[12] Lundberg N. Continuous recording and control of ventricular fluid pressure in neurosurgical practice. Acta Psychiat. Scand. (1960) 36:1–193. 13764297

[13] Julien C. The enigma of Mayer waves: facts and models. Cardiovasc Res. (2006) 70:12–21. 10.1016/j.cardiores.2005.11.008 16360130

[14] Lescot T Naccache L Bonnet MP Abdennour L Coriat P Puybasset L. The relationship of intracranial pressure Lundberg waves to electroencephalograph fluctuations in patients with severe head trauma. Acta Neurochirurgica. (2005) 147:125–9. 10.1007/s00701-004-0355-8 15570441

[15] Momjian S Czosnyka Z Czosnyka M Pickard J. Link between vasogenic waves of intracranial pressure and cerebrospinal fluid outflow resistance in normal pressure hydrocephalus. Br J Neurosurg. (2004) 18:56–61. 10.1080/02688690410001660481 15040716

[16] Spiegelberg A Preuß M Kurtcuoglu V B-waves revisited. Interdiscipl Neurosurg Adv Techniques Case Manag. (2016) 6:13–7. 10.1016/j.inat.2016.03.004

[17] Lalou DA Czosnyka M Donnelly J Lavinio A Pickard JD Garnett M Are Slow Waves of Intracranial Pressure Suppressed by General Anaesthesia? (2018). p. 129–32. 10.1007/978-3-319-65798-1_27 29492547

[18] Balestreri M Czosnyka M Hutchinson P Steiner LA Hiler M Smielewski P Pickard JD. Impact of intracranial pressure and cerebral perfusion pressure on severe disability and mortality after head injury. Neurocritical Care. (2006) 4:8–13. 10.1385/NCC:4:1:008 16498188

[19] Johnston JA. Determinants of mortality in patients with Severe Sepsis. Med Decision Making. (2005) 25:374–86. 10.1177/0272989X05278933 16061889

[20] Miller JD Dearden NM Piper IR Chan KH Control of intracranial pressure in patients with severe head injury. J Neurotrauma. (1992) 9:15–23. 1588623

[21] Sorrentino E Diedler J Kasprowicz M Budohoski KP Haubrich C Smielewski P. Critical thresholds for cerebrovascular reactivity after traumatic brain injury. Neurocritical Care. (2012) 16:258–66. 10.1007/s12028-011-9630-8 21964774

[22] Nourallah B Zeiler FA Calviello L Smielewski P Czosnyka M Menon DK. Critical thresholds for intracranial pressure vary over time in non-craniectomised traumatic brain injury patients. Acta Neurochirurgica. (2018) 160:1315–24. 10.1007/s00701-018-3555-3 29732476 PMC5996002

[23] Schneider G-H Bardt T Lanksch WR Unterberg A. Decompressive craniectomy following traumatic brain injury: ICP, CPP and neurological outcome. In: *Intracranial Pressure and Brain Biochemical Monitoring*. Vienna: Springer (2002). p. 77–9. 10.1007/978-3-7091-6738-0_20 12168363

[24] Sahuquillo J Dennis JA. Decompressive craniectomy for the treatment of high intracranial pressure in closed traumatic brain injury. Cochrane Database Syst Rev. (2019) 12:CD003983. 10.1002/14651858.CD003983.pub3 31887790 PMC6953357

[25] Foreman B Ngwenya LB Stoddard E Hinzman JM Andaluz N Hartings JA. Safety and reliability of bedside, single burr hole technique for intracranial multimodality monitoring in severe traumatic brain injury. Neurocrit Care. (2018) 29:469–80. 10.1007/s12028-018-0551-7 29949001

[26] Maissan IM Dirven PJAC Haitsma IK Hoeks SE Gommers D Stolker RJ. Ultrasonographic measured optic nerve sheath diameter as an accurate and quick monitor for changes in intracranial pressure. J Neurosurg. (2015) 123:743–7. 10.3171/2014.10.JNS141197 25955869

[27] Kristiansson H Nissborg E Bartek J Andresen M Reinstrup P Romner B. Measuring elevated intracranial pressure through noninvasive methods: a review of the literature. J Neurosurg Anesthesiol. (2013) 25:372–85. 10.1097/ANA.0b013e31829795ce 23715045

[28] Hu X Nenov V Bergsneider M Martin N A Data mining framework of noninvasive intracranial pressure assessment. Biomed Signal Process Control. (2006) 1:64–77. 10.1016/j.bspc.2006.05.003

[29] Hughes JA Jackson EC Daley M Modelling intracranial pressure with noninvasive physiological measures. In: *2017 IEEE Conference on Computational Intelligence in Bioinformatics and Computational Biology (CIBCB)*. Manchester (2017). p. 1–8. 10.1109/CIBCB.2017.8058525

[30] Xu P Kasprowicz M Bergsneider M Hu X. Improved noninvasive intracranial pressure assessment with nonlinear kernel regression. IEEE Trans Information Technol Biomed. (2010) 14:971–8. 10.1109/TITB.2009.2027317 19643711 PMC2900395

[31] Sunghan K Scalzo F Bergsneider M Vespa P Martin N Xiao H Noninvasive intracranial pressure assessment based on a data-mining approach using a nonlinear mapping function. IEEE Trans Biomed Eng. (2012) 59:619–26. 10.1109/TBME.2010.2093897 21097375 PMC3130850

[32] Kashif F Verghese G Novak V Czosnyka M Heldt T. Model-based noninvasive estimation of intracranial pressure from cerebral blood flow velocity and arterial pressure. Sci. Transl. Med. (2012) 4:129ra44. 10.1126/scitranslmed.3003249 22496546 PMC4010388

[33] Heldt T Zoerle T Teichmann D Stocchetti N. Intracranial pressure and intracranial elastance monitoring in neurocritical care. Ann Rev Biomed Eng. (2019) 21:523–49. 10.1146/annurev-bioeng-060418-052257 31167100

[34] Joyce C Gorodnitsky I Kutas M. Automatic removal of eye movement and blink artifacts from EEG data using blind component separation. Psychophysiology. (2004) 41:313–25. 10.1111/j.1469-8986.2003.00141.x 15032997

[35] Vigário R Särelä J Jousmäki V Hämäläinen M Oja E. Independent component approach to the analysis of EEG and MEG recordings. IEEE Trans Bio-Med Eng. (2000) 47:589–93. 10.1109/10.841330 10851802

[36] Kuzilek J Kremen V Soucek F Lhotska L. Independent component analysis and decision trees for ECG holter recording de-noising. PLoS ONE. (2014) 9:e98450. 10.1371/journal.pone.0098450 24905359 PMC4048160

[37] Feng M Loy LY Zhang F Guan C. Artifact removal for intracranial pressure monitoring signals: a robust solution with signal decomposition. Proc Ann Int Conf IEEE Eng Med Biol Soc EMBS. (2011) 1:797–801. 10.1109/IEMBS.2011.6090182 22254431

[38] Hüser M. Forecasting intracranial hypertension using time series and waveform features (Master thesis), Zürich: ETH (2015). 10.3929/ethz-a-01049226531851948

[39] Scalzo F Hu X. Semi-supervised detection of intracranial pressure alarms using waveform dynamics. Physiol Measure. (2013) 34:465–78. 10.1088/0967-3334/34/4/465 23524637

[40] Calisto A Galeano M Bramanti A Angileri F Campobello G Serrano S. Analysis of intracranial pressure recordings: comparison of PCA and signal averaging based filtering methods and signal period estimation. Conference Proc. (2010) 2010:3638–41. 10.1109/IEMBS.2010.5627420 21096850

[41] Scalzo F Hamilton R Asgari S Kim S Hu X. Intracranial hypertension prediction using extremely randomized decision trees. Med Eng Phys. (2012) 34:1058–65. 10.1016/j.medengphy.2011.11.010 22401795 PMC3399093

[42] Huang NE Wu ML Qu W Long SR Shen SSP Applications of Hilbert-Huang transform to non-stationary financial time series analysis. Appl Stochastic Models Business Industry. (2003) 19:245–68. 10.1002/asmb.501

[43] Feng M Loy LY Sim K Phua C Zhang F Guan C Artifact correction with robust statistics for non-stationary intracranial pressure signal monitoring. In: *International Conference on Pattern Recognition*. Tsukuba: ICPR (2012). p. 557–60.

[44] Hu X Xu P Lee DJ Vespa P Baldwin K Bergsneider M. An algorithm for extracting intracranial pressure latency relative to electrocardiogram R wave. Physiol Measure. (2008) 29:459–71. 10.1088/0967-3334/29/4/004 18354246 PMC2629794

[45] Megjhani M Alkhachroum A Terilli K Ford J Rubinos C Kromm J. An active learning framework for enhancing identification of non-artifactual intracranial pressure waveforms. Physiol Measure. (2019) 40:015002. 10.1088/1361-6579/aaf979 30562165 PMC6681897

[46] Hu X Xu P Scalzo F Vespa P Bergsneider M. Morphological clustering and analysis of continuous intracranial pressure. IEEE Trans Biomed Eng. (2009) 56:9294. 10.1109/TBME.2008.2008636 19272879 PMC2673331

[47] Lee HJ Jeong EJ Kim H Czosnyka M Kim DJ. Morphological feature extraction from a continuous intracranial pressure pulse via a peak clustering algorithm. IEEE Trans Biomed Eng. (2016) 63:2169–76. 10.1109/TBME.2015.2512278 26841386

[48] Seely AJEJ Macklem PT. Complex systems and the technology of variability analysis. Critical Care. (2004) 8:R367. 10.1186/cc2948 15566580 PMC1065053

[49] Avezaat CJJ Van Eijndhoven JHM Wyper DJ. Cerebrospinal fluid pulse pressure and intracranial volume-pressure relationships. J Neurol Neurosurg Psychiatry. (1979) 42:687–700. 10.1136/jnnp.42.8.687 490174 PMC490301

[50] Teplan M Bajla I Rosipal R Rusnak M. Feature clustering of intracranial pressure time series for alarm function estimation in traumatic brain injury. Physiol Measure. (2017) 38:2015–43. 10.1088/1361-6579/aa8a51 29087961

[51] Mangat HS Chiu Y-L Gerber LM Alimi M Ghajar J Härtl R. Hypertonic saline reduces cumulative and daily intracranial pressure burdens after severe traumatic brain injury. J Neurosurg. (2015) 122:202–10. 10.3171/2014.10.JNS132545 25380107

[52] Kahraman S Dutton RP Hu P Xiao Y Aarabi B Stein DM. Automated measurement of pressure times time dose of intracranial hypertension best predicts outcome after severe traumatic brain injury. J Trauma Injury Infection Critical Care. (2010) 69:110–8. 10.1097/TA.0b013e3181c99853 20038855

[53] Badri S Chen J Barber J Temkin NR Dikmen SS Chesnut RM. Mortality and long-term functional outcome associated with intracranial pressure after traumatic brain injury. Intensive Care Med. (2012) 38:1800–9. 10.1007/s00134-012-2655-4 23011528

[54] Güiza F Depreitere B Piper I Citerio G Chambers I Jones PA. Visualizing the pressure and time burden of intracranial hypertension in adult and paediatric traumatic brain injury. Intensive Care Med. (2015) 41:1067–76. 10.1007/s00134-015-3806-1 25894624

[55] Tian Y Wang Z Jia Y Li S Wang B Wang S. Intracranial pressure variability predicts short-term outcome after intracerebral hemorrhage: a retrospective study. J Neurol Sci. (2013) 330:38–44. 10.1016/j.jns.2013.04.001 23628469

[56] Balestreri M Czosnyka M Steiner LA Schmidt E Smielewski P Matta B. Intracranial hypertension: what additional information can be derived from ICP waveform after head injury? Acta Neurochirurgica. (2004) 146:131–41. 10.1007/s00701-003-0187-y 14963745

[57] Czosnyka M Guazzo E Whitehouse M Smielewski P Czosnyka Z Kirkpatrick P. Significance of intracranial pressure waveform analysis after head injury. Acta Neurochirurgica. (1996) 138:531–42. 10.1007/BF01411173 8800328

[58] Zeiler FA Kim DJ Cabeleira M Calviello L Smielewski P Czosnyka M. Impaired cerebral compensatory reserve is associated with admission imaging characteristics of diffuse insult in traumatic brain injury. Acta Neurochirurgica. (2018) 160:2277–87. 10.1007/s00701-018-3681-y 30251196 PMC6267721

[59] Howells T Lewén A Sköld MK Ronne-Engström E Enblad P. An evaluation of three measures of intracranial compliance in traumatic brain injury patients. Intensive Care Med. (2012) 38:1061–8. 10.1007/s00134-012-2571-7 22527085

[60] Czosnyka M Czosnyka Z Smielewski P. Pressure reactivity index: journey through the past 20 years. Acta Neurochirurgica. (2017) 159:2063–5. 10.1007/s00701-017-3310-1 28849287

[61] Balestreri M Czosnyka M Steiner LA Hiler M Schmidt EA Matta B. Association between outcome, cerebral pressure reactivity and slow ICP waves following head injury. Acta Neurochirurgica Suppl. (2005) 95:25–8. 10.1007/3-211-32318-X_6 16463814

[62] Steiner LA Czosnyka M Piechnik SK Smielewski P Chatfield D Menon DK. Continuous monitoring of cerebrovascular pressure reactivity allows determination of optimal cerebral perfusion pressure in patients with traumatic brain injury. Critical Care Med. (2002) 30:733–8. 10.1097/00003246-200204000-00002 11940737

[63] Aries MJH Czosnyka M Budohoski KP Steiner LA Lavinio A Kolias AG. Continuous determination of optimal cerebral perfusion pressure in traumatic brain injury. Critical Care Med. (2012) 40:2456–63. 10.1097/CCM.0b013e3182514eb6 22622398

[64] Martis RJ Acharya UR Min LC ECG beat classification using PCA, LDA, ICA and Discrete Wavelet Transform. Biomed Signal Process Control. (2013) 8:437–48. 10.1016/j.bspc.2013.01.005

[65] Robertson CS Narayan RK Contant CF Grossman RG Gokaslan ZL Pahwa R. Clinical experience with a continuous monitor of intracranial compliance. J Neurosurgery. (1989) 71:673–80. 10.3171/jns.1989.71.5.0673 2681566

[66] Czosnyka M. Monitoring and interpretation of intracranial pressure. J Neurol Neurosurg Psychiatry. (2004) 75:813–21. 10.1136/jnnp.2003.033126 15145991 PMC1739058

[67] Hall A O'Kane R The best marker for guiding the clinical management of patients with raised intracranial pressure—the RAP index or the mean pulse amplitude? Acta Neurochirurgica. (2016) 158:1997–2009. 10.1007/s00701-016-2932-z 27567609 PMC5025501

[68] Holm S Eide PK. The frequency domain versus time domain methods for processing of intracranial pressure (ICP) signals. Med Eng Phys. (2008) 30:164–70. 10.1016/j.medengphy.2007.03.003 17468029

[69] Sourina O Beng-Ti A Nguyen MK Fractal-based approach in analysis of intracranial pressure (ICP) in severe head injury. Proc 10th IEEE Int Conf Inform Technol Appl Biomed. (2010) 8:1–4. 10.1109/ITAB.2010.5687790

[70] Burr RL Kirkness CJ Mitchell PH. Detrended fluctuation analysis of intracranial pressure predicts outcome following traumatic brain injury. IEEE Trans Biomed Eng. (2008) 55:2509–18. 10.1109/TBME.2008.2001286 18990620 PMC2731428

[71] Soehle M Gies B Smielewski P Czosnyka M. Reduced complexity of intracranial pressure observed in short time series of intracranial hypertension following traumatic brain injury in adults. J Clin Monitor Comp. (2013) 27:395–403. 10.1007/s10877-012-9427-0 23306818

[72] Pincus SM. Approximate entropy as a measure of system complexity. Proc Natl Acad Sci. (1991) 88:2297–301. 10.1073/pnas.88.6.2297 11607165 PMC51218

[73] Hornero R Aboy M Abásolo D McNames J Goldstein B Abasolo D. Interpretation of approximate entropy: analysis of intracranial pressure approximate entropy during acute intracranial hypertension. IEEE Trans Biomed Eng. (2005) 52:1671–80. 10.1109/TBME.2005.855722 16235653

[74] Richman JS Moorman JR. Physiological time-series analysis using approximate entropy and sample entropy. Am J Physiol Heart Circulat Physiol. (2000) 278:H2039–49. 10.1152/ajpheart.2000.278.6.H2039 10843903

[75] Lu C-W Czosnyka M Shieh J-S Smielewska A Pickard JD Smielewski P. Complexity of intracranial pressure correlates with outcome after traumatic brain injury. Brain. (2012) 135:2399–408. 10.1093/brain/aws155 22734128 PMC3407422

[76] Ahmed MU Mandic DP Multivariate multiscale entropy analysis. IEEE Signal Process Lett. (2012) 19:91–4. 10.1109/LSP.2011.2180713

[77] Xu P Scalzo F Bergsneider M Vespa P Chad M Hu X. Wavelet entropy characterization of elevated intracranial pressure. Conference Proc. (2008) 2008:2924–7. 10.1109/IEMBS.2008.4649815 19163318

[78] Xu P Hu X Yao D. Improved wavelet entropy calculation with window functions and its preliminary application to study intracranial pressure. Comp Biol Med. (2013) 43:425–33. 10.1016/j.compbiomed.2013.01.022 23566389

[79] Scalzo F Asgari S Kim S Bergsneider M Hu X. Robust peak recognition in intracranial pressure signals. BioMed Eng Online. (2010) 9:1–19. 10.1186/1475-925X-9-61 20959014 PMC2984490

[80] Xiao H Peng X Asgari S Vespa P Bergsneider M Hu X. Forecasting ICP elevation based on prescient changes of intracranial pressure waveform morphology. IEEE Trans Biomed Eng. (2010) 57:1070–8. 10.1109/TBME.2009.2037607 20659820 PMC2911990

[81] Quachtran B Hamilton R Scalzo F. Detection of intracranial hypertension using deep learning. In: *2016 23rd International Conference on Pattern Recognition (ICPR)*. Cacun (2016). p. 2491–6. 10.1109/ICPR.2016.7900010 28936494 PMC5604755

[82] Scalzo F Liebeskind D Hu X. Reducing false intracranial pressure alarms using morphological waveform features. IEEE Trans Biomed Eng. (2013) 60:235–9. 10.1109/TBME.2012.2210042 22851230 PMC3547536

[83] Hamilton R Xu P Asgari S Kasprowicz M Vespa P Bergsneider M. Forecasting intracranial pressure elevation using pulse waveform morphology. Proc 31st Ann Int Conf IEEE Eng Med Biol Soc. (2009) 2009:4331–4. 10.1109/IEMBS.2009.5332749 19963821

[84] Pimentel MAF Brennan T Lehman L King NKK Ang BT Feng M Outcome prediction for patients with traumatic brain injury with dynamic features from intracranial pressure and arterial blood pressure signals: a Gaussian process approach. J Phys A Math Theor. (2016) 44:85–91. 10.1007/978-3-319-22533-3_17 PMC5484054 27165883

[85] Lazaridis C DeSantis SM Smielewski P Menon DK Hutchinson P Pickard JD. Patient-specific thresholds of intracranial pressure in severe traumatic brain injury. J Neurosurg. (2014) 120:893–900. 10.3171/2014.1.JNS131292 24506248

[86] Güiza F Depreitere B Piper I Van Den Berghe G Meyfroidt G. Novel methods to predict increased intracranial pressure during intensive care and long-term neurologic outcome after traumatic brain injury: development and validation in a multicenter dataset. Critical Care Med. (2013) 41:554–64. 10.1097/CCM.0b013e3182742d0a 23263587

[87] Naraei P Kenez M Sadeghian A A hybrid wavelet based K-means clustering approach to detect intracranial hypertension. IHTC 2017 IEEE Canada Int Humanitarian Technol Conf 2017. (2017) 3:21–5. 10.1109/IHTC.2017.8058190

[88] Czosnyka M Piechnik S Richards HK Kirkpatrick P Smielewski P Pickard JD. Contribution of mathematical modelling to the interpretation of bedside tests of cerebrovascular autoregulation. J Neurol Neurosurgery Psychiatry. (1997) 63:721–31. 10.1136/jnnp636721 9416805 PMC2169860

[89] Hiler M Czosnyka M Hutchinson P Balestreri M Smielewski P Matta B. Predictive value of initial computerized tomography scan, intracranial pressure, and state of autoregulation in patients with traumatic brain injury. J Neurosurg. (2006) 104:731–7. 10.3171/jns.2006.104.5.731 16703877

[90] Gubbi J Buyya R Marusic S Palaniswami M Internet of Things (IoT): a vision, architectural elements, and future directions. Future Gen Comp Syst. (2013) 29:1645–60. 10.1016/j.future.2013.01.010

[91] Jia X Jin C Buzza M Wang W Lee J Wind turbine performance degradation assessment based on a novel similarity metric for machine performance curves. Renew Energy. (2016) 99:1191–201. 10.1016/j.renene.2016.08.018

[92] Kao H-A Jin W Siegel D Lee J A cyber physical interface for automation systems—methodology and examples. Machines. (2015) 3:93–106. 10.3390/machines3020093

[93] Lee J Jin C Bagheri B Cyber physical systems for predictive production systems. Prod Eng. (2017) 11:155–65. 10.1007/s11740-017-0729-4

